# Supplementary material for: Pan‐tissue analysis of allelic alternative polyadenylation suggests widespread functional regulation
Source: Mol Syst Biol. 2020 Apr 20;16(4):e9367. doi: 10.15252/msb.20199367 (PMC7170663; doi:10.15252/msb.20199367)
Supplement: Supplementary file 1 — Expanded View Figures PDF [file MSB-16-e9367-s001.pdf]

Expanded View Figures

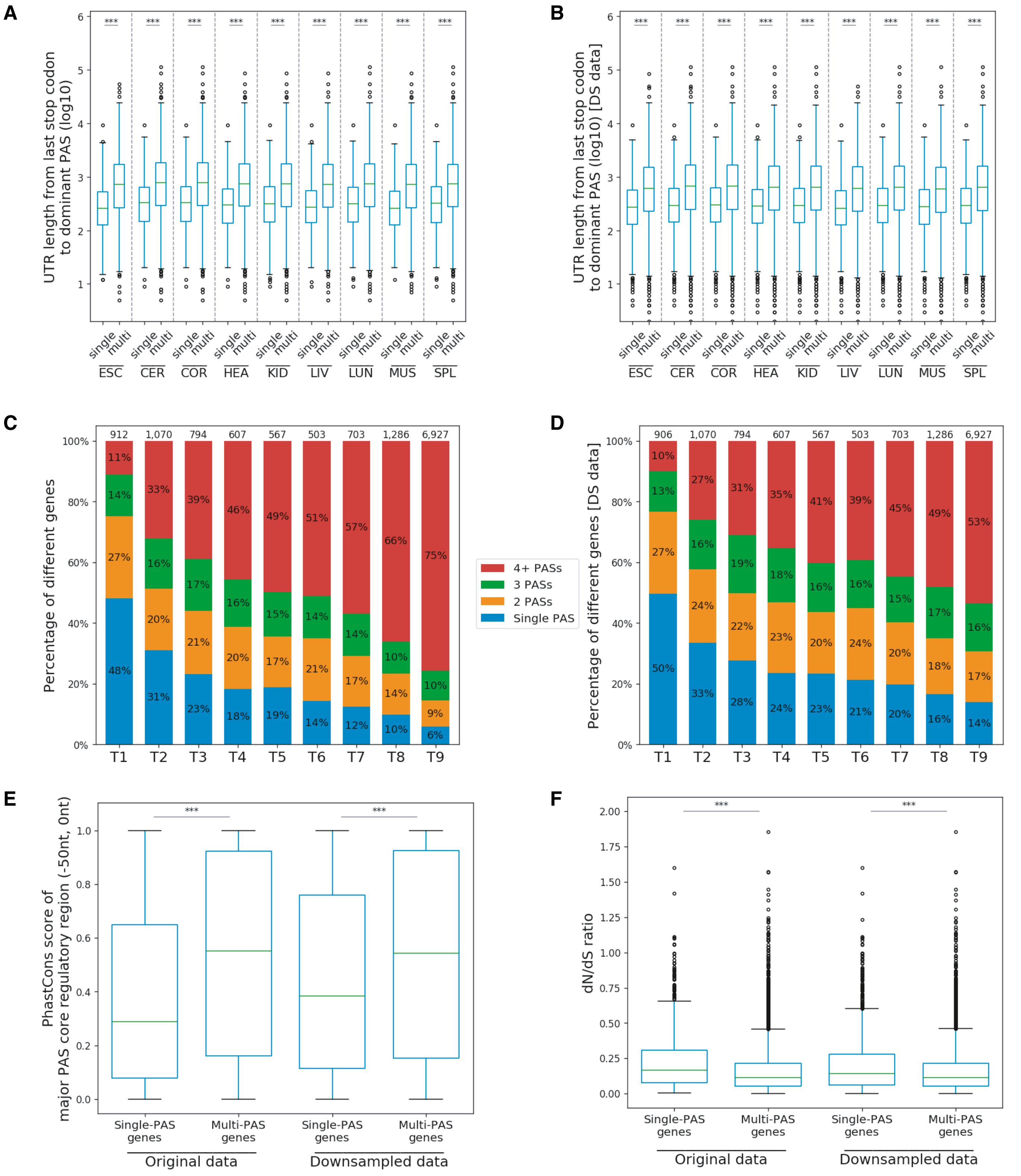

Figure EV1. Feature comparison between single-PAS genes and multi-PAS genes.

A, B Single-PAS genes have shorter 3′UTRs compared to multi-PAS genes in original data (A) and down-sampled data (DS data) (B). The 3′UTR lengths are measured from the last annotated stop codon to the PAS of a single-PAS gene or the dominant 3′UTR-PAS of a multi-PAS gene for each tissue (Mann–Whitney *U*-test, ***$P < 0.001$; ESC: embryonic stem cells, CER: cerebellum, COR: cerebral cortex, HEA: heart, KID: kidney, LIV: liver, LUN: lung, MUS: muscle, SPL: spleen). The boxplots show the Q1 to Q3 quartile values (the box limits), the median (the horizontal green lines), and values within the 1.5 * IQR (the whiskers).

C, D Single-PAS genes are expressed in fewer tissues compared to multi-PAS genes in original data (C) and down-sampled data (D). Tx: x is the number of tissues in which the gene is expressed. The number of genes in each group is indicated above each bar.

E PAS upstream regions in single-PAS genes are less conserved than those of the major PASs in multi-PAS genes. Average PhastCons scores of the *Glires* clade for the core PAS regulatory regions of the single/major PAS are used to estimate sequence conservation (Mann–Whitney *U*-test, ***$P < 0.001$). The boxplot shows the Q1 to Q3 quartile values (the box limits), the median (the horizontal green line), and values within the 1.5 * IQR (the whiskers).

F Single -PAS genes are under weaker selective constraints on protein sequence than multi-PAS genes (Mann–Whitney *U*-test, ***$P < 0.001$). The boxplot shows the Q1 to Q3 quartile values (the box limits), the median (the horizontal green line), and values within the 1.5 * IQR (the whiskers).

 © 2020 The Authors

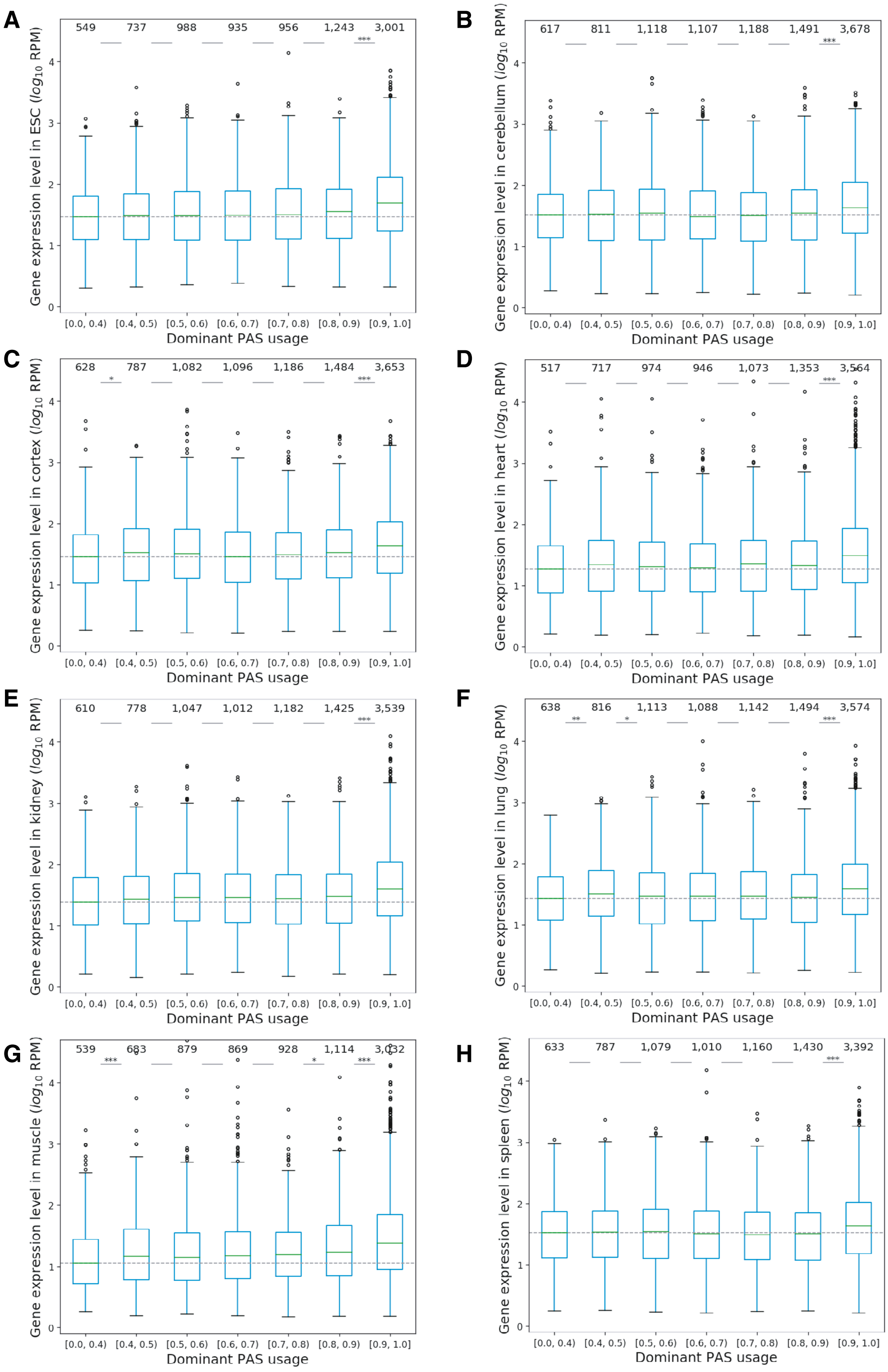

Figure EV2. Multi-PAS genes with higher dominant PAS usage (DPU) express at higher levels in each tissue.

A–H Multi-PAS genes with higher dominant PAS usage (DPU) express at higher levels in ESC (A), cerebellum (B), cortex (C), heart (D), kidney (E), lung (F), muscle (G), and spleen (H) (Mann–Whitney *U*-test, $^{*}P < 0.05$; $^{**}P < 0.01$; $^{***}P < 0.001$). The boxplots show the Q1 to Q3 quartile values (the box limits), the median (the horizontal green lines), and values within the 1.5 * IQR (the whiskers).

© 2020 The Authors

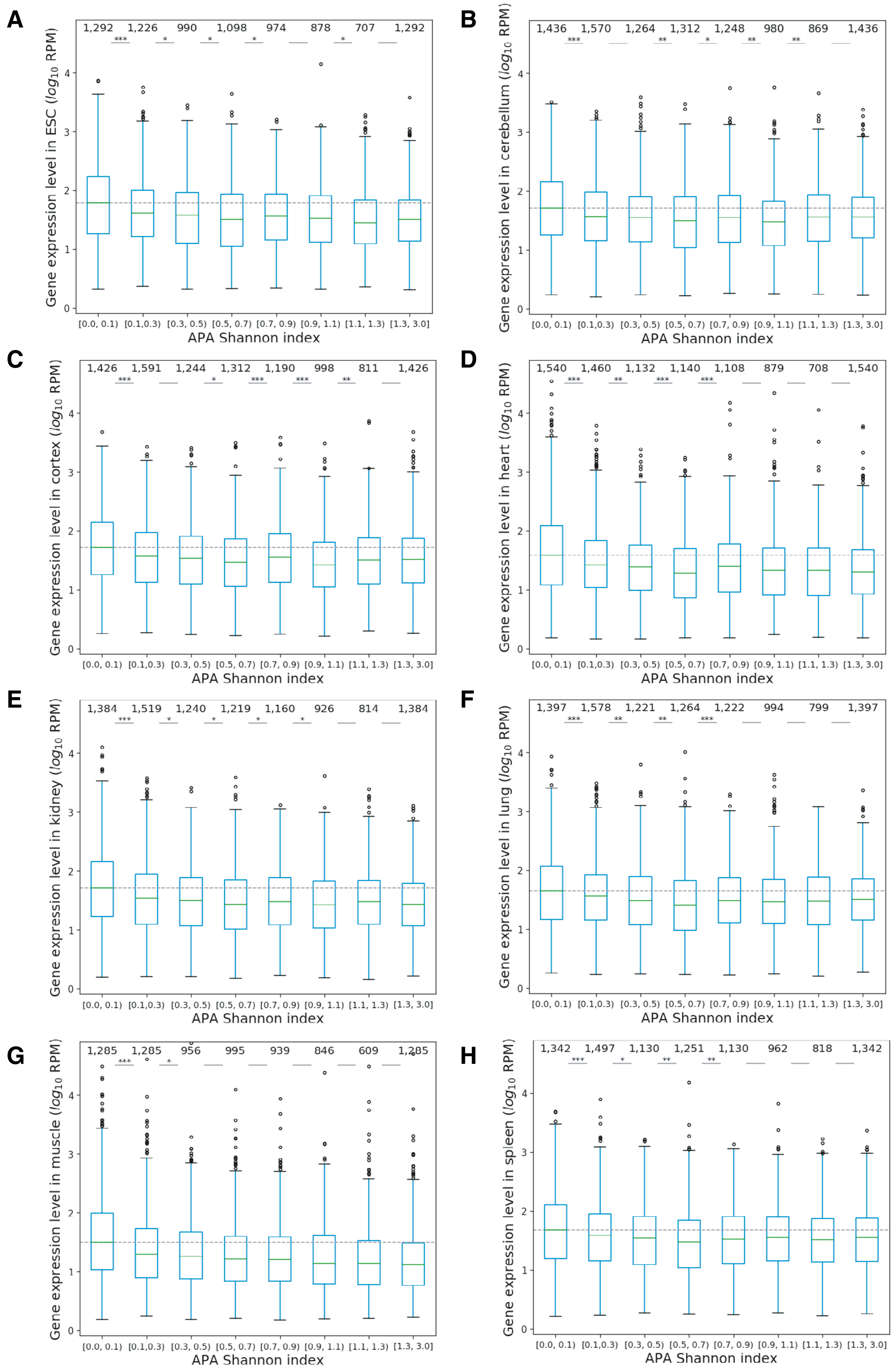

Figure EV3. Multi-PAS genes with lower APA Shannon index express at higher levels in each tissue.

A–H Multi-PAS genes with lower APA Shannon index express at higher levels in ESC (A), cerebellum (B), cortex (C), heart (D), kidney (E), lung (F), muscle (G), and spleen (H) (Mann–Whitney *U*-test, **P* < 0.05; ***P* < 0.01; ****P* < 0.001). The boxplots show the Q1 to Q3 quartile values (the box limits), the median (the horizontal green lines), and values within the 1.5 * IQR (the whiskers).

 © 2020 The Authors

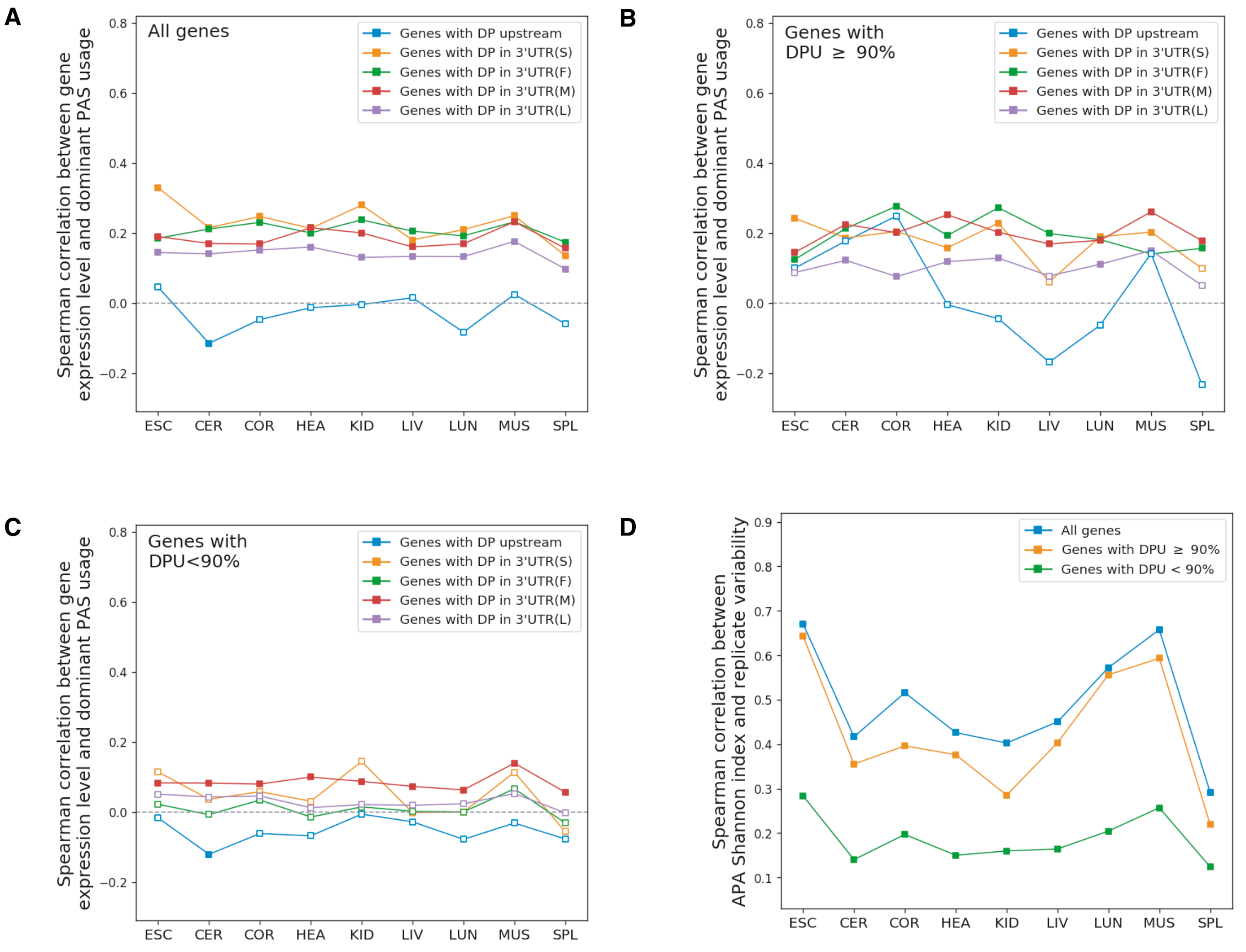

Figure EV4. Spearman correlation between gene expression level and APA diversity.

A–C Spearman correlation between gene expression level and dominant PAS usage for genes with the dominant PAS located in different positions (nine tissues separately). The correlations were calculated from all genes (A), genes with dominant PAS usage equal to 90% or above (B) and genes with dominant PAS usage < 90% (C). An empty square indicates *P* value above 0.01.

D Positive Spearman correlation between APA Shannon index and adjusted APA variability between replicates (ESC: embryonic stem cells, CER: cerebellum, COR: cerebral cortex, HEA: heart, KID: kidney, LIV: liver, LUN: lung, MUS: muscle, SPL: spleen).

© 2020 The Authors

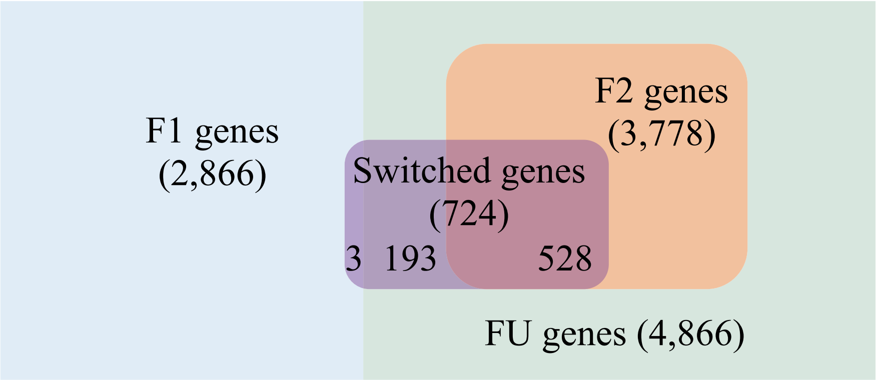

Figure EV5. Schematic Venn diagram of gene groups according to APA within-tissue and across-tissue patterns.

All multi-PAS genes in our test are divided into 2,866 F1 genes (genes with ≥ 90% dominant PAS usage in all expressing tissues), 3,778 F2 genes (genes with usage difference between the second PAS usage and the dominant PAS not larger than 20% in at least one tissue), and 4,866 FU genes (the remaining genes). The 724 switched genes (genes with one PAS exhibiting at least 50% usage difference across tissues) consist of 3 F1 genes, 528 F2 genes, and 193 FU genes. In total, 3,971 genes (34.5%) are observed with two or more isoforms of potentially functional importance, which consist of the 3,778 F2 genes and 193 switched FU genes.

 © 2020 The Authors

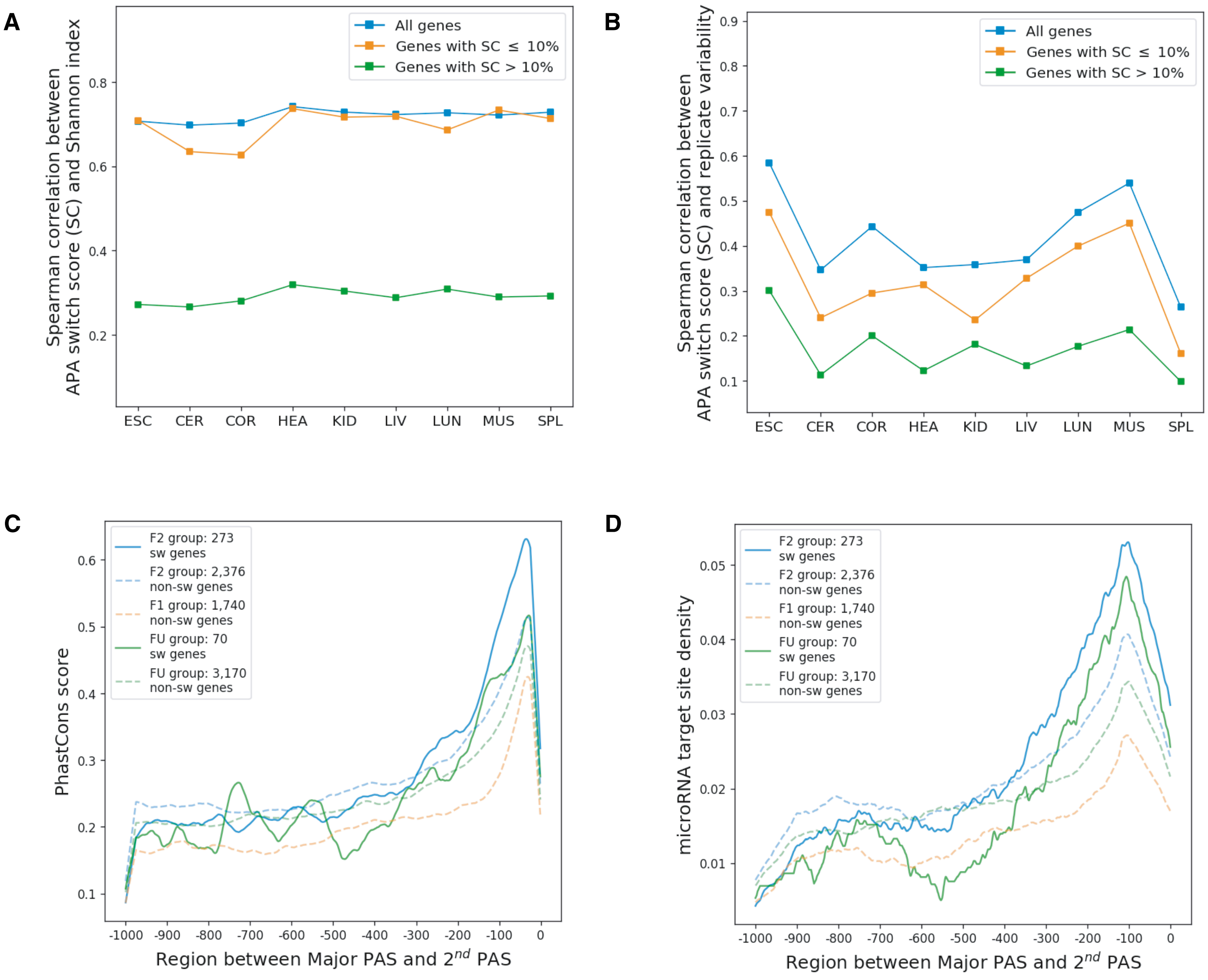

Figure EV6. Features of genes with different switch scores.

A, B Spearman correlation between switch score and Shannon index (A)/APA variability between replicates (B) in each tissue for all expressed genes, genes with switch score (SC) above 10% and genes with switch score equal to 10% or below, respectively.

C, D PhastCons score (C) and microRNA target site density (D) in the region between the major PAS and the 2nd most used PAS of a gene. The solid lines denote switched (sw) genes, while the dashed lines denote non-switched (non-sw) genes.

© 2020 The Authors

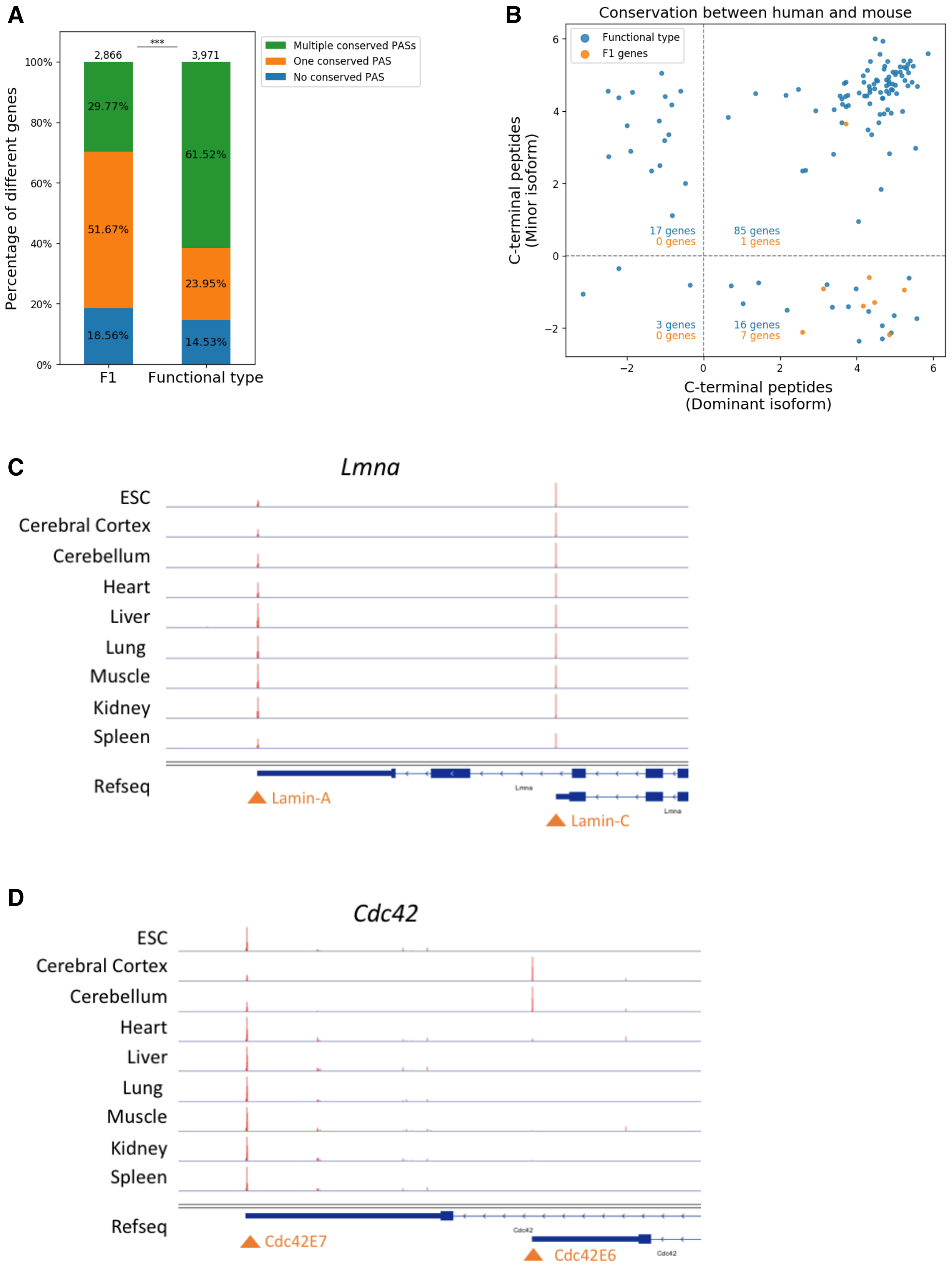

Figure EV7. Conservation of PAS and the c-terminal peptides encoded by APA isoforms.

A Fraction of genes with different numbers of PASs conserved between humans and mouse in F1 and APA functional type genes (Fisher exact test for fraction of genes with multiple conserved PASs: ***$P < 0.001$).

B Comparison of c-terminal peptide conservation between F1 and APA functional type genes in which APA of the two conserved PASs involves alternative splicing of the last exon (see Materials and Methods). Dominant isoforms are encoded specifically by the dominant APA isoforms, while minor isoforms are by the 2nd most used isoforms. The numbers in different quadrants indicate the number of genes in corresponding quadrants (blue for APA functional type genes and orange for F1 genes).

C, D 3′mRNA-seq track for *Lmna* (C) and *Cdc42* (D) across nine tissues/cells.

 © 2020 The Authors

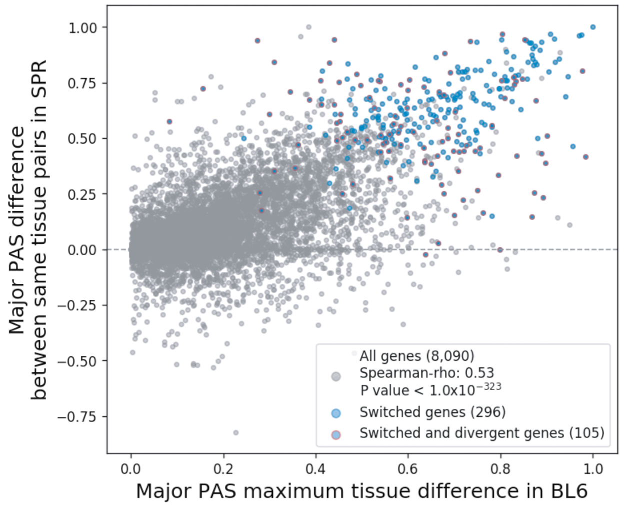

Figure EV8. Allelic major PAS usage difference with different switch scores.

Scatterplot showing the maximum major PAS usage difference in the BL6 allele across tissues and the major PAS usage difference in the SPR allele between the same tissue pairs.

© 2020 The Authors
