# Supplementary material for: Pan‐tissue analysis of allelic alternative polyadenylation suggests widespread functional regulation
Source: Mol Syst Biol. 2020 Apr 20;16(4):e9367. doi: 10.15252/msb.20199367 (PMC7170663; doi:10.15252/msb.20199367)
Supplement: Supplementary file 2 — Table EV1 [file MSB-16-e9367-s002.docx]

**Table EV1:** Sequencing and mapping statistics

| Tissues | Sequencing reads | Uniquely mapped reads to F1 genome | Reads mapped to annotated PAS | Reads assigned to alleles | Uniquely mapped reads assigned to BL6 | Uniquely mapped reads assigned to SPR |
| --- | --- | --- | --- | --- | --- | --- |
| ESC_rep1 | 38,310,730 | 23,751,837 | 15,947,315 | 7,412,455 | 3,882,991 | 3,529,464 |
| ESC_rep2 | 35,091,726 | 22,394,965 | 15,005,588 | 7,498,364 | 3,934,259 | 3,564,105 |
| ESC_rep3 | 21,681,581 | 15,338,107 | 10,446,957 | 4,914,733 | 2,573,618 | 2,341,115 |
| Cerebellum_rep1 | 54,505,336 | 36,195,485 | 16,255,602 | 6,886,854 | 3,475,496 | 3,411,358 |
| Cerebellum_rep2 | 62,398,509 | 41,470,979 | 20,299,148 | 9,556,173 | 4,803,324 | 4,752,849 |
| Cerebellum_rep3 | 49,281,118 | 32,405,466 | 15,724,700 | 7,052,146 | 3,633,479 | 3,418,667 |
| Cortex_rep1 | 51,514,545 | 29,840,400 | 15,199,678 | 6,325,032 | 3,199,478 | 3,125,554 |
| Cortex_rep2 | 47,556,684 | 31,571,326 | 14,930,980 | 6,899,155 | 3,474,974 | 3,424,181 |
| Cortex_rep3 | 60,813,146 | 41,415,226 | 21,981,871 | 10,146,001 | 5,261,683 | 4,884,318 |
| Heart_rep1 | 39,198,588 | 27,717,264 | 18,155,775 | 9,303,786 | 4,567,363 | 4,736,423 |
| Heart_rep2 | 37,603,240 | 25,237,822 | 16,492,023 | 7,774,114 | 3,843,454 | 3,930,660 |
| Heart_rep3 | 41,424,038 | 28,660,969 | 17,738,042 | 8,490,478 | 4,299,328 | 4,191,150 |
| Kidney_rep1 | 35,937,583 | 26,829,182 | 16,433,110 | 8,653,710 | 4,377,486 | 4,276,224 |
| Kidney_rep2 | 37,532,445 | 26,426,207 | 16,212,675 | 8,665,554 | 4,360,660 | 4,304,894 |
| Kidney_rep3 | 42,986,429 | 32,883,284 | 19,859,440 | 10,412,048 | 5,340,977 | 5,071,071 |
| Liver_rep1 | 35,273,651 | 24,650,841 | 16,809,143 | 8,539,118 | 4,433,304 | 4,105,814 |
| Liver_rep2 | 34,539,447 | 23,674,845 | 16,465,203 | 8,270,815 | 4,158,050 | 4,112,765 |
| Liver_rep3 | 38,616,805 | 27,977,797 | 18,834,656 | 9,362,280 | 4,691,604 | 4,670,676 |
| Lung_rep1 | 39,569,519 | 30,034,230 | 17,944,172 | 7,402,410 | 3,872,079 | 3,530,331 |
| Lung_rep2 | 42,860,286 | 31,327,479 | 18,004,532 | 7,768,580 | 3,991,192 | 3,777,388 |
| Lung_rep3 | 44,752,188 | 32,989,887 | 19,089,545 | 8,401,752 | 4,442,926 | 3,958,826 |
| Muscle_rep1 | 33,885,070 | 25,768,338 | 17,804,093 | 8,893,782 | 4,612,756 | 4,281,026 |
| Muscle_rep2 | 35,618,364 | 28,209,331 | 18,196,292 | 9,550,882 | 4,892,245 | 4,658,637 |
| Muscle_rep3 | 37,610,304 | 28,760,316 | 19,131,365 | 9,624,880 | 5,182,837 | 4,442,043 |
| Spleen_rep1 | 45,270,912 | 27,529,476 | 15,088,769 | 6,540,080 | 3,302,392 | 3,237,688 |
| Spleen_rep2 | 45,438,308 | 33,121,258 | 18,709,988 | 8,252,102 | 4,157,270 | 4,094,832 |
| Spleen_rep3 | 42,932,761 | 30,359,884 | 16,237,077 | 7,305,359 | 3,795,632 | 3,509,727 |
| Average | 41,933,456 | 29,131,193 | 17,148,064 | 8,144,542 | 3,882,991 | 3,529,464 |
