# Supplementary material for: Pan‐tissue analysis of allelic alternative polyadenylation suggests widespread functional regulation
Source: Mol Syst Biol. 2020 Apr 20;16(4):e9367. doi: 10.15252/msb.20199367 (PMC7170663; doi:10.15252/msb.20199367)
Supplement: Supplementary file 3 — Table EV2 [file MSB-16-e9367-s003.docx]

**Table EV2:** Spearman correlation between APA diversity (Shannon index) and dN/dS ratio in different tissues.

| Shannon index vs. dN/dS ratio | Spearman’s rho | | | *P* value | | |
| --- | --- | --- | --- | --- | --- | --- |
|  | All genes | Genes with DPU ≥ 90% | Genes with DPU < 90% | All genes | Genes with DPU ≥ 90% | Genes with DPU < 90% |
| ESC | -0.02 | 0.00 | -0.01 | 0.11 | 0.92 | 0.40 |
| Cerebellum | 0.02 | -0.01 | 0.01 | 0.05 | 0.51 | 0.70 |
| Cortex | 0.02 | -0.01 | 0.01 | 0.02 | 0.62 | 0.42 |
| Heart | -0.01 | -0.01 | -0.01 | 0.21 | 0.56 | 0.46 |
| Kidney | -0.01 | -0.01 | -0.01 | 0.19 | 0.76 | 0.62 |
| Liver | -0.03 | -0.00 | -0.02 | 0.01 | 0.92 | 0.29 |
| Lung | -0.02 | -0.03 | -0.03 | 0.09 | 0.06 | 0.06 |
| Muscle | -0.03 | -0.00 | -0.01 | 0.02 | 0.91 | 0.69 |
| Spleen | -0.02 | -0.01 | -0.01 | 0.09 | 0.67 | 0.31 |
